# Short-Term Cardiac Autonomic Recovery after a Repeated Sprint Test in Young Soccer Players

**DOI:** 10.3390/sports7050102

**Published:** 2019-04-30

**Authors:** Cesar Cavinato Cal Abad, Lucas Adriano Pereira, Vinicius Zanetti, Ronaldo Kobal, Irineu Loturco, Fabio Yuzo Nakamura

**Affiliations:** 1 Nucleus of High Performance, São Paulo, SP 04753-070, Brazil; lucasa_pereira@outlook.com (L.A.P.); rokorin2002@hotmail.com (R.K.); irineu.loturco@terra.com.br (I.L.); 2 Red Bull Brazil, Jarinu, SP 13240-000, Brazil; vinicius.zanetti@redbull.com; 3 Department of Physical Education, Federal University of Paraiba; João Pessoa, PB 58051-900, Brazil; fabioy_nakamura@yahoo.com.br

**Keywords:** heart rate variability, young players, training load monitoring

## Abstract

The aim of this study was to describe the time course (within 2 h post-exercise) of heart rate variability (HRV) recovery following a traditional repeated sprint ability (RSA) test applied to youth soccer players. Twenty-four young soccer players (18.4 ± 0.5 years) undertook the following assessments: (1) 10 min rest in the seated position for HRV assessment; (2) a repeated sprint ability (RSA) test; (3) passive recovery in the seated position for 10 min, immediately after finishing the RSA test and 1 h and 2 h post-RSA test. During the HRV measurements (using the natural log of root mean square difference of successive normal RR intervals—lnRMSSD) the participants were instructed to assume a comfortable sitting position, remaining awake and breathing spontaneously for 10 min. Magnitude-based inference was used in the analyses. After the RSA test, the post-1 h measure was almost certainly lower than the resting measure, but almost certainly higher than the lnRMSSD measured post-RSA test. The lnRMSSD post-2 h was likely lower than the resting lnRMSSD and very likely higher than post-1 h. In conclusion, lnRMSSD is severely depressed after performing an RSA test, and reactivation is incomplete after 2 h of passive recovery. This result should be considered by practitioners when applying successive training sessions within intervals shorter than 2 h.

## 1. Introduction

Repeated sprints are commonly implemented in team sports to assess and train top-level athletes [1,2]. It has been shown that fatigue during repeated sprints ensues due to metabolic and neuromuscular factors [3]. This leads to significant physiological perturbation, performance impairments and signs of muscle damage that can persist from hours to days [4]. In addition, the cardiac autonomic activity as measured by the vagal-related heart rate variability (HRV) index (i.e., the natural log of root mean square difference of successive normal RR intervals—lnRMSSD) is substantially depressed immediately after finishing a training session including repeated sprints (in the first 10 min of recovery) but increased after 24 h of recovery [5,6,7,8,9,10].

HRV is an inexpensive, time-efficient, and non-invasive method to assess the status of the autonomic nervous system [11]. HRV measurements may be obtained either during rest conditions or after submaximal tests, and their acute responses have been widely used to monitor training load, prescribe workout sessions, and predict sports performance [12,13,14]. HRV includes important information concerning the physiological recovery processes after training stimulus and serve as an indicator of appropriate physiological condition for training [15]. Previous studies suggest that training prescriptions guided by HRV recovery result in greater improvements in physical fitness than predefined (i.e., non-tailored) training prescriptions [16,17,18,19]. However, the HRV is generally obtained in the morning, after waking, in subjects who train once a day. Therefore, in athletes who train twice a day, the behavior of HRV between two consecutive exercise sessions may be crucial to optimize training responses and reduce the risk of maladaptation.

Soccer is an intermittent and high-intensity team sport which depends of both aerobic and anaerobic fitness [20]. To develop soccer-related capabilities such as endurance, strength, and linear and change of direction speed, elite players usually train twice a day, interspersed by lower than 24 h of recovery. However, the short-term time course (i.e., HRV variations within the same day) of cardiac autonomic responses after the RSA test remains unknown. As such, the understanding of the cardiac stress and recovery cycle after typical training or testing practices (e.g., repeated sprint ability; [RSA]) may help practitioners to better and more effectively prescribe exercise sessions, based on individual needs and profiles.

In this regard, considering the HRV responses after RSA testing is especially important because post-exercise HRV is associated with the exercise anaerobic contribution [5] and the circulating stress system metabolites [12]. HRV recovery dynamics also reflect training intensity and status [21], and high resting vagally-mediated HRV indicates the individual predisposition to properly cope with some specific physical and technical demands and, as a consequence, improve performance [15]. That said, it seems that HRV could be a useful tool for monitoring the athletes’ recovery status, as this indicates their readiness for subsequent training stimuli [22].

To our knowledge, no study has attempted to examine the short-term cardiac autonomic recovery after a typical RSA test in young soccer players. The aim of this study was to describe the time course (within 2 h post-exercise) of HRV recovery following a traditional RSA test applied to soccer players. It was hypothesized that the lnRMSSD would be fully reestablished compared to the resting value after 2 h of recovery, indicating their readiness for the next training session.

## 2. Materials and Methods

The present study was conducted during the 1st week of a specific preparatory period of a high-level under-20 male soccer team in the lead-up to the principal Brazilian national championship of the category. The tests and measures were assessed on Monday morning at 10:00 a.m. The players were instructed to maintain their normal routine concerning sleeping, food and fluid intake and to avoid alcohol and caffeine consumption and strenuous physical activity for 24 h prior to the tests. The assessments were performed as follows: (1) 10 min rest in the seated position for HRV assessment; (2) RSA test; (3) passive recovery in the seated position for 10 min, immediately after finishing the RSA test and 1 h, and 2 h post-RSA test. During the HRV measurements (e.g., rest, post-RSA, and 1 h and 2 h post-RSA) the participants were advised to assume a comfortable sitting position, remain awake and breathe spontaneously for 10 min. The players performed the RSA test wearing heart rate monitors in order to guarantee the rapidness of the post-exercise measurements. The resting HRV, and the recovery immediately post-RSA test were performed in quiet outdoor facilities. The HRV recovery after 1 h and 2 h post-RSA was assessed in quiet indoor facilities. The HRV measure was limited to 2 h post-exercise due to the training schedule of the investigated team. After this time, technical-tactical training was administered by the coaches. The average temperature during the data acquisition was ≈26 °C.

### 2.1. Participants

Twenty-four young soccer players (18.4 ± 0.5 years; 70.1 ± 8.4 kg; 175.5 ± 7.3 cm) volunteered to participate in the study. All procedures received clearance from the local research ethics committee and were conducted in accordance with the ethical standards of the Declaration of Helsinki. The participants and their legal guardians (for the <18-year-old players) signed an informed consent form before participation. All players were familiarized with the RSA test and HRV recording procedures prior to commencement of the investigation. The study was approved by a local ethics committee, and the ethical code is 39658514.8.0000.5493.

### 2.2. HRV Analysis

Players were fitted with the elastic straps and the R–R data were recorded using the Team2 System (Polar Electro, Kupio, Finland). Occasional ectopic beats were automatically replaced with interpolated adjacent R–R interval values. The lnRMSSD was calculated during the final 5 min of RR intervals out of the 10 min recording period in the following conditions: rest; immediately post-RSA test; and 1 h and 2 h after the RSA test. During the recovery period the athletes remained in resting conditions in their accommodation and were oriented to avoid stress situations and physical efforts until the next HRV recording.

### 2.3. Repeated Sprint Ability Test

The RSA test was performed on a synthetic soccer field and consisted of 6 × 40 m shuttle sprints (20 + 20 m) interspersed with 20 s of passive recovery [23]. The athletes started from a line set 50 cm behind the photocell (Smart Speed; Fusion Sport, Coopers Plains, Australia), sprinted for 20 m, touched a line with one foot, and returned to the starting line as fast as possible. After 20 s of passive recovery, the soccer player started again. Five seconds before commencing each sprint, the subjects assumed the ready position and waited for the start signal [23]. The total duration of the RSA test was less than 2 min.

### 2.4. Statistical Analysis

Data are presented as mean ± standard deviation (SD). To test the differences between the resting HR and HRV, and the different recovery periods, magnitude-based inference (MBI) was used [24]. The probabilities of finding lower/trivial/higher values compared to the resting HRV, and the planned comparisons between different recovery periods were assessed qualitatively as follows: <1%, almost certainly not; 1% to 5%, very unlikely; 5% to 25%, unlikely; 25% to 75%, possible; 75% to 95%, likely; 95% to 99%, very likely; >99%, almost certain. If the chances of having higher and lower results were both >5%, the true difference was assessed as unclear. To assess the MBI data an Excel spreadsheet (Microsoft^®^, Inc, Redmond, WA, USA) was used. The respective spreadsheet is available at: http://www.sportsci.org/index.html.

## 3. Results

**Figure 1:**
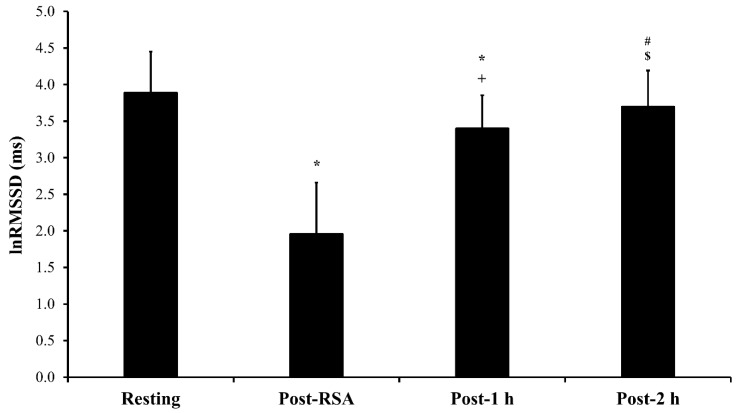
Resting heart rate variability and post-exercise heart rate variability recovery at the different times evaluated. * Almost certainly lower (100/00/00) compared to resting; # Likely lower (82/18/00) compared to resting; + Almost certainly higher (00/00/100) compared to Post-RSA. $ Very likely higher (00/01/99) compared to post-1 h.

The RSA_best_ and RSA_mean_ were 6.90 ± 0.20-s and 7.25 ± 0.19-s, respectively. Figure 1 presents the comparisons of lnRMSSD between the different periods. After the RSA test, the post-1 h measure was almost certainly lower than the resting measure, but almost certainly higher than the lnRMSSD measured post-RSA test. The lnRMSSD post-2 h was likely lower than the resting lnRMSSD and very likely higher than post-1 h.

## 4. Discussion

This study was the first to show HRV recovery immediately (within 10 min) and remotely (up to 2 h) after an RSA test. The test format used herein is typically undertaken to assess and train soccer players [23,25]. In addition, after 1 h of passive recovery, the soccer players demonstrated substantially increased cardiac vagal activity (73.7% increment, compared to immediately post-RSA test), although not attaining the resting levels. Finally, after 2 h, there was an additional vagal reactivation compared to post-1 h of recovery (8.75% increment, compared to post-1-h), while the lnRMSSD at this moment was still lower than the resting value. Thus, the HR response reflected the HRV dynamics.

The most important finding of this study was that the vagal-related HRV remained below the resting condition after 2 h, meaning that the cardiac autonomic suppression was expressive and long lasting. Such prolonged cardiac autonomic depression was surprising due to the extremely low duration of the protocol (<3 min). In the study of Seiler et al. [21], for example, after 6 × 3 min at a velocity eliciting 95–100% of maximal oxygen consumption with 2 min of recovery, the root mean square difference of successive normal RR intervals (RMSSD) returned to the resting values after only ≈ 60 min, in endurance runners. It appears that the greater anaerobic contribution and elevation in circulating stress system metabolites caused by the RSA test [5] were able to disturb the cardiac autonomic system more severely than a session of interval training, especially when considering the remote phases of the post-exercise recovery (in our case, 1 h and 2 h). This agrees with other studies that found a reduction on HRV after short high intensity and supramaximal exercises [5,6,7,8,9,21,26,27,28].

It has been shown that total cardiac autonomic recovery from highly anaerobic exercise tasks (two Wingate tests interspersed with 3 min recovery) is incomplete after 1 h but complete 24 h later [28]. The duration of optimal post-repeated sprint recovery to allow total restoration of HRV and the possible effects of training [27] and fitness status [21] on this outcome remain to be investigated. In addition, the effectiveness of acute interventions to accelerate HRV restoration (e.g., cold water immersion) [29,30,31] should be tested after RSA tests or training sessions.

Despite a fast HRV recovery would be expected after a RSA test, in fact it does not occur. Coaches and physical conditioners should pay attention on athletes´ HRV recovery even after a short (but intense) training/testing sessions. Indeed, if consecutive training session are applied without an adequate HRV recovery, the athletes may show an incomplete cardiac autonomic restoration which, chronically, could signaling a possible unfavorable training response [32,33]. Therefore, our results highlight the importance of HRV monitoring specially if consecutive training sessions will be applied with an incomplete HRV recovery.

This study is limited by the absence of a control group (or condition) and by the duration of remote time course of HRV recovery (limited to 2 h, due to the training schedule of the team). Indeed, investigating longer recovery periods for parasympathetic reactivation would require a control group (or condition) because HRV varies throughout the day due to the circadian rhythm [34] and could bias the interpretation of the changes in lnRMSSD. Nevertheless, the design used in this investigation reflects the actual situation of many types of team sports, which regularly perform two consecutive training sessions per day.

In conclusion, lnRMSSD is severely depressed after performing an RSA test and reactivation is incomplete after 2 h of passive recovery. In practical terms, team sports coaches and strength and conditioning trainers should consider the implications of prescribing loads after an incomplete HRV recovery from the previous session. It seems that longer recovery periods are necessary to fully enable the recovery of the cardiac autonomic function after a short-duration all-out stimulus. Accordingly, since muscle fatigue was not considered in the present study other functional, biochemical, and subjective measures to establish the optimal recovery duration remain to be investigated, together with HRV in future investigations.
